# Efficacy and Safety of Pramipexole Sustained Release versus Immediate Release Formulation for Nocturnal Symptoms in Chinese Patients with Advanced Parkinson's Disease: A Pilot Study

**DOI:** 10.1155/2021/8834950

**Published:** 2021-03-03

**Authors:** Haiyan Zhou, Shuhua Li, Hongmei Yu, Shenggang Sun, Xinhua Wan, Xiaodong Zhu, Chun-Feng Liu, Ling Chen, Wei Xiang, Yaqing Sun, Haibo Chen, Shengdi Chen

**Affiliations:** ^1^Department of Neurology, Ruijin Hospital, Shanghai Jiao Tong University School of Medicine, Shanghai, China; ^2^Department of Neurology, Beijing Hospital, National Center of Gerontology, Beijing, China; ^3^Department of Neurology, Institute of Geriatric Medicine, Chinese Academy of Medical Science, Beijing, China; ^4^Department of Neurology, The First Hospital of China Medical University, Shenyang, China; ^5^Department of Neurology, Wuhan Union Hospital, Wuhan, China; ^6^Department of Neurology, Peking Union Medical College Hospital, Beijing, China; ^7^Department of Neurology, Tianjin Medical University General Hospital, Tianjin, China; ^8^Department of Neurology and Suzhou Clinical Research Center of Neurological Disease, The Second Affiliated Hospital of Soochow University, Suzhou, China; ^9^Department of Neurology, The First Affiliated Hospital, Sun Yet-Sen University, Guangzhou, China; ^10^Boehringer Ingelheim (China), Shanghai, China

## Abstract

**Objective:**

To explore the efficacy and safety of pramipexole sustained release (SR) versus pramipexole immediate release (IR) in treating nocturnal symptoms in levodopa-treated Chinese patients with advanced Parkinson's disease (PD) and sleep disturbances.

**Method:**

SUSTAIN was an open-label, randomised, active-controlled parallel group exploratory pilot study (NCT03521635). A total of 98 patients were randomly allocated (1 : 1) to either pramipexole SR (*n* = 49) or pramipexole IR (*n* = 49) groups. The primary endpoint was a change from baseline in PD Sleep Scale 2^nd^ version (PDSS-2) total score at 18 weeks. A reduction in score represents improvement. Secondary endpoints included Nocturnal Hypokinesia Questionnaire, Scales for Outcomes in PD Sleep Scale, Early Morning Off (EMO), Epworth Sleepiness Scale, PD Questionnaire-8, and responder rates as measured by PDSS-2 total score (<18), EMO scores (≥1 point change), Clinical Global Impression Improvement scale, and Patient Global Impression-Improvement scale. Other endpoints included motor complications (MDS-UPDRS part IV) score. Adverse events were evaluated for each group.

**Results:**

The mean pramipexole dose for both groups was 1.5 mg/day at week 18, and the mean changes in PDSS-2 total score for pramipexole SR and IR were –13.7 (95% CI –16.0 to –11.4) and –14.4 (–16.8 to –12.0) (difference of 0.7; *p*=0.688). Change from baseline for both groups achieved the minimal clinical important difference threshold (MCID = –3.44). No significant difference was observed in change from baseline for other measures of sleep-related disturbances or responder rates. For motor complications, a greater improvement in MDS-UPDRS part IV score was observed in pramipexole SR over IR (–3.4 vs –2.3; treatment group difference: –1.1; *p*=0.036). Both groups had comparable safety profiles.

**Conclusion:**

In Chinese patients with advanced PD and sleep disturbances, pramipexole SR and IR have similar benefits in the treatment of nocturnal symptoms and safety, and an improvement from baseline in nocturnal symptoms was observed regardless of pramipexole formulation.

## 1. Introduction

### 1.1. Background

Parkinson's disease (PD) is a chronic, irreversible, degenerative disorder of the central nervous system, with slowly progressive degeneration of the nigrostriatal dopaminergic systems [1]. The management of PD in advanced stages aims to reduce and/or control functional disability and improve quality of life [2, 3]. Sleep disturbances occur in 40% to 98% of PD patients [4–6] and can adversely affect both patients and caregivers [7].

The multifactorial and multidimensional nature of sleep disturbances precludes the use of a single instrument to measure sleep; specific and validated scales are needed to capture the complexity of the disease. Parkinson's Disease Sleep Scale (PDSS) 2^nd^ version (PDSS-2) has been shown to be a reliable, valid, and promising tool to measure treatment response for nocturnal disabilities and sleep disorders in PD [8]; a total PDSS-2 score of ≥18 indicates severe sleep disturbances [9]. Various other tools to capture night sleep and day-time sleepiness have been used and reviewed [10–13].

Pramipexole is a nonergot dopamine agonist approved as monotherapy in early PD as well as adjunct therapy to levodopa in advanced PD [14]. Pramipexole sustained release (SR) is a new once-daily formulation of pramipexole, which has shown similar efficacy, safety, and tolerability to those of immediate release (IR) pramipexole taken three times daily. Compared with the IR formulation, a continuous delivery of treatment through pramipexole SR over an extended period [15] especially at night may be beneficial in treating nocturnal symptoms associated with PD.

The main objective of the study was to explore the efficacy between pramipexole SR versus pramipexole IR on nocturnal symptoms (as measured by the change from baseline to 18 weeks in PDSS-2 score) in levodopa-treated patients with advanced PD.

## 2. Methods

### 2.1. Study Design

The SUSTAIN (Sustained and immediate-release pramipexole on the noctUrnal Symptoms of paTients with Advanced ParkInsoN's disease) was a phase IV, multicentre, open-label, randomised, active-controlled parallel group exploratory pilot study. Patients were recruited from 12 sites (with screened subjects) across China between 17 August 2018 and 2 September 2019.

Patients were randomly allocated (1 : 1) to receive either pramipexole SR or pramipexole IR, available as 0.375 mg or 0.75 mg tablets for the SR formulation and 0.25 mg or 1.0 mg for the IR formulation. The randomisation was performed by Boehringer Ingelheim Pharma GmbH & Co. KG, Germany, and the randomisation list was generated using a validated system and verified by a trial-independent statistician. An external vendor implemented the assignment based on the randomisation list. According to the summary of product characteristics for pramipexole SR and IR [16, 17], the treatment dose was uptitrated over 7 weeks, based on efficacy (Patient Global Impression-Improvement (PGI-I) scale) and tolerability of study medication, until optimal daily dose was reached (according to investigator judgement). The dose of study medication should be up titrated in all patients who are not at least “a little better” on the PGI-I and reduced to previous dose in case of dopaminergic side effects. Patients then entered a maintenance phase of 11 weeks at optimised dosage. The dose reduction phase after completion of maintenance phase lasted up to 7 days unless treatment was to be continued, as judged by investigators. During the 18-week active treatment phase, the timing of administration as well as the doses of these treatments will remain consistent for each patient.

The study was conducted in accordance with the principles of the Declaration of Helsinki and the Harmonized Tripartite Guideline for Good Clinical Practice from the International Council for Harmonization and was approved by local authorities and ethics committees at each participating site. The study is registered with ClinicalTrials.gov (NCT03521635). All patients provided written informed consent.

### 2.2. Patients

Patients included were male or female of ≥30 years old, diagnosed with advanced PD with at least 2 years of disease history and disease stages II–IV at on-time based on the modified Hoehn & Yahr scale confirmed by at least bradykinesia and one of the following signs: resting tremor and/or rigidity. The patients were also required to present with motor fluctuations, have clinically relevant sleep disturbances as measured by PDSS-2 (total score of ≥18) and sleep-related difficulties at night (scoring ≥2 on the PDSS-2 question 9) or morning (i.e., the frequency of “feeling like bodily movements are poor when you wake up?” was at least 2 to 3 days during the past week), and were receiving levodopa treatment. Continuing use of other anti-Parkinsonian agents was allowed, provided the dosage was unchanged during the prior 4 weeks and remained unchanged during study treatment phase. Patients were excluded if any of the following were reported: atypical Parkinsonian syndromes, any type of dementia (assessed by the Mini-Mental State Examination using a cut-off of 24) or psychiatric disorder, any history of deep brain stimulation, psychiatric or non-PD medical disorders capable of impeding trial participation, clinically significant hypotension or electrocardiographic abnormalities (>2 ULN levels on liver blood tests, or creatinine clearance <50 mL/min), any use of night-time SR dopaminergic (anti-PD) drug, hypnotic, neuroleptics, atypical antipsychotic, or stimulants within the prior 4 weeks, use of flunarizine within 3 months prior to randomisation visit, or serious sleep apnea hypopnea syndrome (scoring ≥3 on the PDSS-2 question 15). Details of the inclusion and exclusion criteria are available in Supplementary Table S1.

### 2.3. Study Assessments/Outcomes

Efficacy endpoints were evaluated by calculating the change from baseline at week 18 in the following outcome measures and then comparing the difference between the pramipexole SR and IR groups. The primary endpoint evaluated was sleep disturbance, assessed using the PDSS-2 total score. The difference in change from baseline for primary endpoint was assessed with a prespecified minimal clinically important difference (MCID = –3.44) [9]. Subgroup analyses for the primary endpoint were done for age (<65, ≥65 years), pramipexole final dose (low/0.375–1.5 mg/day, medium/2.25–3.0 mg/day, high/3.75–4.5 mg/day), Hoehn & Yahr stage (on-phase: 2, 2.5, 3, 4), activities of daily living severity at baseline (MDS-UPDRS part II: high/>12 points, low/≤12 points), complication severity at baseline (MDS-UPDRS IV: high/>4 points, low/≤4 points), disease severity at baseline (off-time according to patient diary: high/≥4 hours, low/<4 hours), baseline levodopa dose (≤400 mg, >400 mg), and baseline equivalent levodopa dose (≤400 mg, >400 mg).

The secondary efficacy endpoints included other measures of night- and day-time disturbances related to sleep, including the Nocturnal Hypokinesia Questionnaire (NHQ), Scales for Outcomes in Parkinson's Disease-Sleep Scale (SCOPA-S), Early Morning Off (EMO), and Epworth Sleepiness Scale (ESS). Quality of life in patients with PD as measured by the Parkinson's Disease Questionnaire-8 (PDQ-8) and responder rates of patients showing improvement in PDSS-2 total score (<18 points), EMO scores (≥1 point change), Clinical Global Impression-Improvement (CGI-I) scale, and PGI-I scale were also assessed.

Further efficacy endpoints were impact on motor experiences of daily living and motor complications as measured by the Movement Disorder Society-Sponsored Revision of the Unified Parkinson's Disease Rating Scale (MDS-UPDRS) part II and part IV; other measures of psychological health and quality of life included the General Geriatric Depression Scale- (GDS-) 15 score and EQ-5D-5L, healthcare resource utilization (HCRU) and costs (including direct/indirect medical and direct non-medical costs) incurred by patients during the course of study, and also Caregiver Burden Inventory (CBI).

A detailed description of the efficacy outcome measures is given in Table 1 for the primary and secondary endpoints and Supplementary Table S2 for further endpoints. Safety was assessed based on the physical examination, weight, vital signs, 12-lead electrocardiogram, laboratory tests, adverse events (AEs), serious AEs, and the Modified Minnesota Impulsive Disorders Interview (MMIDI).

### 2.4. Statistical Analyses

As a pilot study, the total eligible sample size of 80 was determined based on trial feasibility instead of statistical power for hypothesis testing. The randomised sample size of 86 considered a drop-out rate of 5%.

Demographic and safety data were analysed descriptively on all patients who received at least one dose of either pramipexole SR or IR (treated set, TS). The efficacy was analysed on all randomised patients who received pramipexole SR or IR with a baseline and at least one PDSS-2 total score measurement in maintenance period (full analysis set, FAS). For the primary endpoint, mean changes from baseline were analysed using a mixed model with repeated measures (MMRM) with treatment, visit in the maintenance period, and treatment-by-visit interaction, and analysis for primary endpoint was performed including visits in both titration and maintenance periods. Secondary and further endpoints were analysed as per primary endpoint, with respective baseline as covariate. For responder rates, logistic regression analyses were performed with treatment and baseline (if baseline was measured) as the independent variables. Additional post hoc analyses were performed to investigate: change from baseline in MDS UPDRS-IV by items (4.1–4.6) and by subgroups (age, PD duration, L-Dopa equivalent dose at baseline, degree of time spent with dyskinesia and in off state), and change from baseline in CBI subscales (details in supplementary materials). Given the exploratory nature of this study, no adjustments were made for multiplicity for all comparisons and overall false positive risk might be inflated. The data were analysed using Statistical Analysis System (SAS) for Windows version 9.4.

## 3. Results

### 3.1. Patient Disposition and Baseline Characteristics

**Figure 1:**
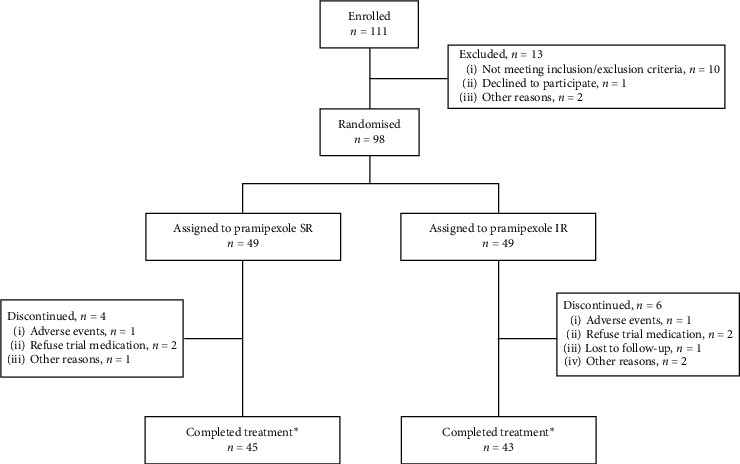
Flow chart: enrolment, treatment allocation, and completion of study participants. ^*∗*^Completed titration/maintenance. IR, immediate release; SR, sustained release.

A total of 98 patients were enrolled and equally assigned to pramipexole SR (*n* = 49) or IR (*n* = 49) groups, respectively (Figure 1). Of these, four patients in the SR group and six patients in the IR group prematurely discontinued during the titration/maintenance phase. For each of the treatment group, two patients discontinued due to “refused to continue taking trial medication” and one patient due to AEs. One patient in the IR group was lost to follow-up. A total of three patients (one in IR group, two in SR group) discontinued due to other reasons. Both groups had similar baseline clinical and demographic characteristics (mean age, 61.0 ± 9.8 years; Hoehn & Yahr stages II-III; duration of disease, 5.0 ± 3.4 years; mean total PDSS-2, 28.5 ± 7.6 points) (Table 2).

### 3.2. Efficacy

**Figure 2:**
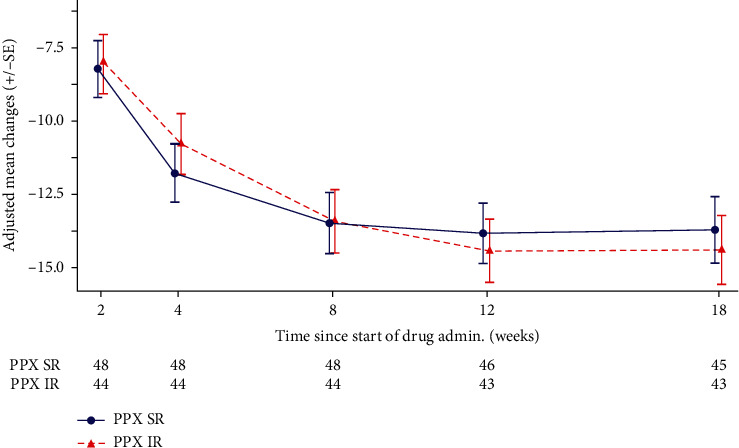
Adjusted mean changes from baseline in PDSS-2 over time in titration/maintenance period (FAS). Adjusted with factors treatment, titration/maintenance period, and covariate baseline. The current trend is consistent with the planned primary analysis for mean changes from baseline in PDSS-2 over time, which adjusted with factors treatment, maintenance phase and covariate baseline. Unstructured covariance matrix, Kenward–Roger approximation for denominator degrees of freedom. FAS, full analysis set; IR, immediate release; PDSS-2, Parkinson's Disease Sleep Scale 2^nd^ version; PPX, pramipexole; SE, standard error; SR, sustained release.

The adjusted mean changes from baseline at week 18 in PDSS-2 total score for pramipexole SR and IR were –13.7 (95% CI –16.0 to –11.4) and –14.4 (95% CI –16.8 to –12.0), respectively (Table 3). The difference between treatment groups was not statistically significant for PDSS-2 total score (treatment group difference: 0.7, 95% CI –2.7 to 4.0; *p*=0.688) and subscales (Table 3). The magnitude of treatment difference did not achieve the prespecified MCID (≤–3.44), but the change from baseline was <–3.44 for both pramipexole SR and IR; this was contributed by mean reductions from baseline in all PDSS-2 total score subgroups (disturbed sleep, motor symptoms at night, PD symptoms at night; Table 3). The adjusted mean changed from baseline in PDSS-2 total score over the titration/maintenance period (Figure 2). The result for changes in PDSS-2 total score at week 18 from baseline by predefined subgroups is available in Supplementary Table S3. It was noted that the change at week 18 in PDSS-2 total score from baseline was numerically larger in patient subgroups with higher baseline Hoehn & Yahr stage on phase and higher complication severity.

**Figure 3:**
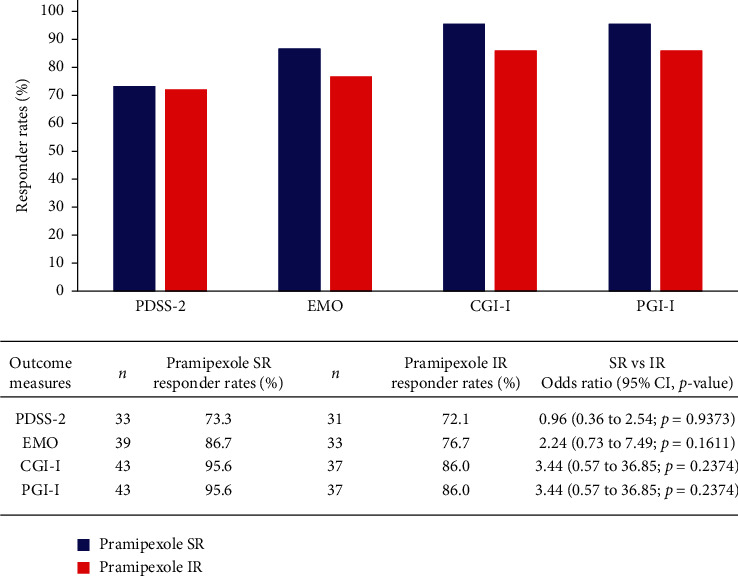
Responder rates in patients (FAS). The responder rates for each outcome measure: PDSS-2, total score <18; EMO, patient improved at least 1 comparing to his/her baseline condition; CGI-I, clinician rated of any improvement (1 = very much better, 2 = much better, or 3 = a little better); PGI-I, patient rated of any improvement (1 = very much better, 2 = much better, or 3 = a little better). CGI-I, Clinical Global Impression of Improvement; CI, confidence interval; EMO, Early Morning Off; FAS, full analysis set; IR, immediate release; PDSS-2, Parkinson's Disease Sleep Scale-2; PGI-I, Patient Global Impression of Improvement; SR, sustained release.

No significant treatment differences were observed for adjusted mean change from baseline at week 18 in all secondary endpoints, that is, NHQ (SR versus IR: –1.9 versus –1.7), SCOPA-S night-time sleep (–3.8 versus –3.2), SCOPA-S day-time sleepiness (–1.4 versus –0.9), EMO (–1.8 versus –1.5), and ESS (–2.4 versus –2.6). There were a similar proportion of patients in both the pramipexole SR and IR groups that showed improvements in responder rates analyses (PDSS-2, EMO, CGI-I, and PGI-I) (Figure 3) and quality of life PDQ-8.

No significant treatment differences in adjusted mean change from baseline at week 18 were observed for further endpoints MDS-UPDRS II, GDS-15, EQ-5D-5L, and CBI (Supplementary Table S4). Both treatment groups reported improvements in MDS-UPDRS part IV (SR versus IR: –3.4 [95% CI –4.1 to –2.7] versus –2.3 [95% CI –3.0 to –1.6]), with greater improvements observed with pramipexole SR (treatment group difference: –1.1, *p*=0.036). Additional post hoc analyses showed a generally consistent trend in favor of pramipexole SR for MDS-UPDRS part IV items and selected subgroups (Supplementary Table S5 and S6). Within the CBI subscales, the difference between caregivers of patients who received pramipexole SR (*n* = 13) versus IR (*n* = 11) was greatest for physical burden (–3.7 versus –1.0) (Supplementary Table S5). The median (Q1-Q3) hours spent by caregivers during day-time and night-time were 0 (0–4) hours versus 0 (0–8) hours and 0 (0–1.5) versus 0 (0–0) hours for pramipexole SR and IR groups, respectively. No pattern of differences between treatment groups were observed for HCRU and costs.

### 3.3. Study Drug

The mean exposure to the study drug in the pramipexole SR and IR treatment groups was similar (121.2 versus 115.8 days); at 18 weeks, mean pramipexole dose for SR and IR groups was 1.5 mg/day and mean cumulative dose was 162.1 mg and 161.0 mg, respectively. Both treatment groups achieved good overall compliance (80%–120% compliance: 100% for pramipexole SR and 95.9% for pramipexole IR).

### 3.4. Safety Outcomes

The proportion of AEs for both groups were similar, with almost half of the patients in each group reporting at least one AE (Table 4). Most of the AEs were of mild or moderate intensity. One patient who received pramipexole IR reported a serious AE; none were reported for the pramipexole SR group. This patient with a serious AE experienced mechanical ileus, cholecystitis, cholelithiasis, and femoral neck fracture; these were assessed by the investigator as not related. Treatment-related AEs were reported in 32.7% and 38.8% of the SR and IR group, respectively. Of impulse control related AEs, two patients reported compulsive sexual behaviour and one experienced visual hallucinations (SR group); one patient reported compulsive shopping and one reported gambling (IR group). Overall, both formulations of pramipexole were generally well tolerated with similar safety profiles.

## 4. Discussion

SUSTAIN is the first open-label, randomised, active-controlled study to compare the efficacy, and safety of pramipexole SR versus pramipexole IR in treating nocturnal symptoms in patients with advanced PD receiving levodopa. After 18 weeks, both treatment groups demonstrated similar benefits for the primary endpoint, that is, change from baseline in PDSS-2 total score, and secondary endpoints which include other measures of night- and day-time disturbances related to sleep. Based on the suggested and prespecified MCID for PDSS-2 [9], the observed changes from baseline in both pramipexole SR and IR suggest clinical effects of improvement in nocturnal symptoms regardless of formulations.

Sleep disturbance is a common nonmotor symptom in PD [18, 19] and may worsen with increased disease duration [20]. Nocturnal akinesia and other motoric disorders of sleep contribute to motor-related sleep disturbances in Parkinson's disease [18]. Altogether, patients with poor sleep, interacting with motor dysfunction and mood disorders, are at risk of reduced quality of life [21–23]. The dopaminergic system is associated with both sleep and wakefulness; therefore, PD treatment (i.e., levodopa and/or dopamine agonist) which influences the dopaminergic system could potentially have an effect on sleep [20]. Existing studies of dopaminergic medications suggest benefits of pharmacological treatment on sleep disturbances, including those of extended-release formulation [8, 24, 25]. This is supported by the Chinese consensus for the management of sleep disturbances in patients with PD, which recommend the use of dopamine agonists for treatment of insomnia [6].

In a retrospective exploratory analysis of advanced PD patients with sleep disturbances while on stable levodopa, a numerical benefit in sleep was observed with pramipexole SR over pramipexole IR [26]. Using an open-label, randomised parallel group design, this 18-week study did not demonstrate significant differences between pramipexole formulations on PDSS-2 total score and subscales. Given the long terminal phase half-life of pramipexole IR (8–12 hours) [27], the multiple dosing might result in steady-state concentration including night-time and contributed to the obvious improvement of nocturnal motor disturbances. Therefore, even if the SR formulation can improve the 24 h fluctuations of drug plasma concentrations [28], both formulations might have reached a similar ceiling effect for the efficacy of nocturnal symptoms. In terms of change from baseline in PDSS-2 total score, both pramipexole SR and IR treatment still achieved the MCID threshold of –3.44 [29]; while lacking a placebo control group, these improvements in sleep were consistent with that observed in the previous study of pramipexole versus placebo [25]. Similarly in a randomised controlled study of rotigotine, the mean PDSS-2 total score decreased by –5.9 points with 24-h rotigotine (baseline, 19.3) and by –1.9 points with placebo (baseline, 20.5) after 4 weeks of treatment [30].

Long-term therapy with levodopa is associated with the development of motor fluctuations and dyskinesia [31]; dopamine agonists such as pramipexole that have longer half-lives have demonstrated a reduction in rate of development of dyskinesia than levodopa [32]. It was noted that both treatment groups in this pramipexole study reported clinical improvements in motor complications compared with baseline (MCID = –0.9) [33] after 18 weeks, with greater improvements observed in pramipexole SR; the same numerical trend was observed for all the individual UPDRS IV items, particularly in terms of time spent with dyskinesia. Nonetheless, these analyses are post hoc without adjustment on multiplicity and hence should be interpreted with caution. These results are consistent with the pramipexole IR long-term outcome study in advanced PD [34]. Meanwhile, depression is also commonly associated with poor sleep [22, 35]. Sleep disturbance and depression may overlap and arise from common neurobiological networks, yet both may differentially contribute to quality of life [36].

While the pilot data presented suggests no difference in sleep outcomes between pramipexole SR or IR, the practical approach of a once-daily therapy may still offer greater convenience benefit and facilitate better treatment adherence [37]. Noncompliance in PD treatment management has costly effects on patients' health and healthcare systems [38], especially among the elderly with other medical comorbidities and complex medication regimes [39]. Caregivers can play an important role in the regular care and treatment adherence [40], and poor quality of sleep in PD patients has been associated with depression and increased burden in caregivers [41, 42]. Therefore, the engagement and support for the well-being of caregivers should be a necessary part of the PD treatment system.

The AE data during the study for both groups were comparable. Few patients who were treated with either pramipexole formulations experienced dizziness (6.1%) or nausea (4.1%) in this study; these were lower compared with the AE profile in a similar study of rotigotine (10% and 21%) [30]. Serious AEs and AEs leading to discontinuation were minimal in both pramipexole groups, suggesting that the AEs and titration of treatment were well-managed and patients continued to show improvements in motor complications.

Interpretation of the findings in SUSTAIN is limited by its sample size, open-label design, and lack of placebo arm to demonstrate treatment effectiveness. Self-reported data are inherently vulnerable to response bias [43] and objective measures of sleep. For example, polysomnography (gold standard) or triaxial accelerometer was not used in this study as these measures require time and cost and are labour-intensive. Nevertheless, this is the first study to evaluate difference in the efficacy of the SR versus IR formulation in nocturnal symptoms for PD. The study applied a formal cut-off of ≥18 points on the PDSS-2 total score identifying those with existing sleep disturbances and allocated patients to different treatment arms in a randomised manner. PDSS-2 is a validated measure of sleep disturbances in PD [8]; however, given the complex and multidimensional aspect of poor sleep, a comprehensive battery of self-report measures of day- and night-time sleep disturbances was applied, including independent measures of patient's night-time symptoms captured by caregivers (NHQ by caregivers), which patients' themselves might not be aware of.

## 5. Conclusions

In Chinese patients with advanced PD and sleep disturbances, pramipexole SR and IR have similar benefits in the treatment of nocturnal symptoms and safety; and an improvement from baseline in nocturnal symptoms was observed regardless of pramipexole formulation.

## Figures and Tables

**Table 1:** Description of outcome measures for primary and secondary efficacy endpoints.

Outcome measures	Description
Primary efficacy endpoint	
Parkinson's disease Sleep Scale 2^nd^ version (PDSS-2)	The PDSS-2 consists of 15 questions about various sleep and nocturnal disturbances which are to be rated by the patients using one of five categories, from 0 (never) to 4 (very often). Patients were asked to rate the severity of each question based on their experience during the past week (7 days) from 0 (never) to 4 (very often, that meant 6 to 7 days a week). PDSS-2 total score ranges from 0 (no disturbance) to 60 (maximum nocturnal disturbance). There are three sub-scales from the three-factor solution, one comprising nocturnal movement-related problems (factor 1: “motor problems at night”), a second describing the disease specific symptoms (factor 2: “PD symptoms at night”) and the third representing sleep specific disturbances (factor 3: “Disturbed sleep”). A total score of ≥18 defines clinically relevant PD-specific sleep disturbances. According to previous study, any improvements in PDSS-2 less than −3.44 points could represent clinically important changes for the patients. That was to say, a minimal clinically important difference (MCID) of PDSS-2 [horvath et al. 2015] was -3.44 points.

Secondary efficacy endpoints	
Nocturnal Hypokinesia Questionnaire (NHQ)	The Nocturnal Hypokinesia Questionnaire is designed to assess hypokinesia symptoms in night in PD patients, composed of two sections. Section 1 is assessed by PD patients. There are four domains in section 1 to assess “turning over in bed,” “getting out of bed,” “Parkinsonian motor symptoms,” and “others” separately. The domains in Section 2 are the same with it in Section 1. Section 2 is assessed by spouses or caregivers who are with the patients during the night.
Scales for Outcomes in Parkinson's Disease (SCOPA)-Sleep	The SCOPA-Sleep is a short, self-rating scale designed to evaluate nocturnal sleep quality and day-time sleepiness in patients with PD. It is composed of three parts: a night-time scale (NS), a single item about perceived quality of nocturnal sleep, and a day-time sleepiness scale (DS) that includes an item about unexpected onset of sleep.
	The NS is a five-item scale with four response options (0-not at all to 3-very much) that addresses nighttime disturbances that “occurred in the previous month.” The five items include sleep initiation, sleep fragmentation, sleep efficiency, sleep duration, and early wakening. Total NS score runs from 0 to 15, with higher scores reflecting more severe problems. The additional “quality of sleep” question assesses the overall night-time sleep quality on a seven-point scale (ranging from slept very well to slept very badly). This item is not included in the total NS score. The DS subscale evaluates day-time sleepiness, also in the past month, and includes six items with four response options, from 0 (never) to 3 (often), and a maximum total score of 18. These DS items were addressed as to how often the patient had fallen asleep unexpectedly, but in particular situations (while sitting quietly, watching TV or reading, and talking to someone) had had difficulty to remain awake and self-perception of day-time sleepiness as a problem.
Early Morning Off (EMO)	The EMO is measured by the question of “do you feel like your bodily movements are poor when you wake up?” Patients answered this question according to the frequency during the previous one week by scoring from 0 (“never”) to 4 (“very often” or “6 to 7 days a week”). EMO score had to be rated before the first anti-PD drug taking in early morning, and within half an hour after waking up. The responder of EMO was the patient improved at least 1 comparing to his/her baseline condition.
Epworth Sleepiness Scale (ESS)	The ESS is a patient-rated scale about how likely one is to fall asleep during situations as passive and inconsequential as “watching TV” to as active as “sitting and talking to someone” and as consequential as “in a car, while stopped for a few minutes in traffic.” “Chance of dozing” is rated as an integer from 0 (no chance) to 3 (high).
Parkinson's disease Questionnaire (PDQ) -8	The PDQ-39 is a self-administered, disease specific measure of health status which covers eight dimensions of ill health and contains 39 questions. The 8 domains include mobility, activities of daily living, emotional well-being, stigma, social support, cognition, communication, and bodily discomfort.
	The PDQ-8 is an 8-item self-report questionnaire derived from PDQ-39. Each item selected is the one most highly correlated with the overall domain score to which it contributes. The items are summed together and transformed onto a score from 0 to 100. The PDQ-8 has been shown to exhibit appropriate levels of reliability, validity, and responsiveness.
Clinical Global Impression of Improvement (CGI-I)	The CGI was developed by the Early Clinical Drug Evaluation Unit (ECDEU) of the National Institute of Mental Health as an independent, simple way for clinicians to make overall evaluations of a patient's central nervous system (CNS) disease status. The ratings were used initially in outpatients with various psychiatric disorders. The CGI-I was rated (from 1: very much improved, to 7: very much worse) by the same evaluator to assess the overall status of Parkinson's disease, after interviewing the patient about the various aspects of the PD and after evaluating AE and concomitant treatments. The evaluator completed the scale by comparing the patients' status during the past week to their baseline condition.
	The responder of CGI-I was the clinician rated of any improvement (1 = very much better, 2 = much better, or 3 = a little better).
Patient Global Impression of Improvement (PGI-I)	The PGI-I scale is a patient-rated instrument which was used to measure the improvement (from 1: Very much better, to 7: Very much worse) of the patient's Parkinson disease symptoms throughout the study. Patients completed the scale by comparing their status during the past week to their baseline condition. The responder of PGI-I was the patient rated of any improvement (1 = very much better, 2 = much better, or 3 = a little better).

**Table 2:** Demographics and baseline characteristics (TS).

	Pramipexole SR (*N* = 49)	Pramipexole IR (*N* = 49)
Male, *n* (%)	28 (57.1)	31 (63.3)
Age (years), mean (SD)	61.1 (10.8)	60.9 (8.8)
Age (years), min-max	31–79	42–79
BMI (kg/m^2^), mean (SD)	22.8 (3.4)	23.9 (3.0)
Disease duration (years), mean (SD)	4.9 (3.7)	5.1 (3.0)
Hoehn & Yahr stage II-III on time, *n* (%)	49 (100.0)	49 (100.0)
PDSS-2 total score, mean (SD)	27.8 (6.5)	29.2 (8.5)
MMSE score, mean (SD)	28.5 (1.6)	27.9 (1.8)
UPDRS part II total score, mean (SD)	16.6 (5.75)	17.2 (5.65)
UPDRS part IV total score, mean (SD)	7.5 (2.86)	6.6 (2.61)
Levodopa daily dosage, mg, mean (SD)	383.2 (158.1)	444.4 (169.3)
Levodopa equivalent dose, mg, mean (SD)	555.0 (264.1)	594.9 (263.4)
Concomitant PD therapies^*∗*^, *n* (%)	49 (100.0)	49 (100.0)
Dopa and dopa derivatives	49 (100.0)	49 (100.0)
Adamantane derivatives	12 (24.5)	15 (30.6)
MAO-B inhibitors	16 (32.7)	11 (22.4)
Tertiary amines^+^	3 (6.1)	5 (10.2)
Dopamine agonist	0 (0.0)	1 (2.0)^§^
Other dopaminergic agents^‡^	5 (10.2)	2 (4.1)

^*∗*^Patients must not have been treated with dopamine agonists within 4 weeks prior to randomisation visit. A concomitant treatment with one or more of the following drugs will be allowed (at a stable dose for at least 4 weeks prior to randomisation visit and the investigator does not intend to change this treatment during the treatment phase): anti-Parkinsonian anticholinergics, selegiline, rasagiline, or other MAO-B-Inhibitor, amantadine, or entacapone (or other COMT-inhibitor). ^+^Tertiary amines included the preferred name “trihexyphenidyl hydrochloride” and “trihexyphenidyl.” ^‡^Other dopaminergic agents included the preferred name “entacapone.” ^§^One patient in the pramipexole IR group was treated with dopamine agonist (piribedil) which was prohibited according to the exclusion criteria. The patient was documented as an important protocol deviation and excluded from the sensitivity analysis. BMI, body mass index; IR, immediate release; MAO-B, monoamine oxidase type B; MMSE, Mini-Mental State Examination; PD, Parkinson's disease; PDSS-2, Parkinson's Disease Sleep Scale 2^nd^ version; SD, standard deviation; SR, sustained release; TS, treated set.

**Table 3:** Adjusted mean changes in primary and secondary efficacy endpoints at week 18 from baseline (FAS).

	Pramipexole SR				Pramipexole IR				SR vs IR	
	*N*	Baseline, mean (SD)	Week 18, mean (SD)	Change^*∗*^, mean (SE); 95% CI	*N*	Baseline, mean (SD)	Week 18, mean (SD)	Change^*∗*^, mean (SE); 95% CI	Adjusted mean difference (SE)	95% CI, *p* value
PDSS-2 total score	45	28.0 (6.47)	14.6 (7.27)	–13.7 (1.16); –16.0 to –11.4	43	29.3 (8.32)	14.3 (9.19)	–14.4 (1.20); –16.8 to –12.0	0.7 (1.68)	–2.7 to 4.0; *p*=0.688
PDSS-2 total score—disturbed sleep	45	13.1 (3.18)	7.8 (3.54)	–5.4 (0.55); –6.5 to –4.3	43	13.8 (3.54)	7.6 (4.25)	–5.9 (0.57); –7.0 to –4.7	0.4 (0.79)	–1.1 to 2.0; *p*=0.589
PDSS-2 total score—motor symptoms at night	45	8.3 (3.52)	3.7 (3.23)	–4.7 (0.45); –5.6 to –3.8	43	8.9 (4.02)	3.8 (3.34)	–4.8 (0.47); –5.7 to –3.9	0.1 (0.65)	–1.2 to 1.4; *p*=0.844
PDSS-2 total score—PD symptoms at night	45	6.7 (2.51)	3.0 (2.52)	–3.7 (0.39); –4.4 to –2.9	43	6.7 (2.79)	2.9 (3.17)	–3.7 (0.41); –4.6 to –2.9	0.1 (0.57)	–1.0 to 1.2; *p*=0.865
NHQ by patient	45	5.6 (1.70)	3.7 (2.50)	–1.9 (0.35); –2.6 to –1.2	43	5.9 (1.84)	4.1 (2.66)	–1.7 (0.36); –2.4 to –0.9	–0.2 (0.51)	–1.2 to 0.8; *p*=0.690
NHQ by caregivers	13	5.2 (2.09)	4.0 (2.58)	–1.4 (0.61); –2.7 to –0.2	11	6.1 (1.81)	5.3 (2.10)	–0.6 (0.67); –2.0 to 0.8	–0.8 (0.91)	–2.7 to 1.1; *p*=0.376
SCOPA—night-time	45	7.9 (3.03)	4.2 (2.89)	–3.8 (0.46); –4.7 to –2.9	43	8.2 (2.85)	4.9 (3.46)	–3.2 (0.47); –4.1 to –2.2	–0.6 (0.66)	–1.9 to 0.7; *p*=0.333
SCOPA —overall night-time sleep quality	45	4.7 (1.19)	3.0 (1.33)	–1.9 (0.20); –2.3 to –1.5	43	4.9 (1.23)	3.2 (1.46)	–1.6 (0.21); –2.0 to –1.2	–0.3 (0.29)	–0.8 to 0.3; *p*=0.374
SCOPA —day time	45	3.7 (2.63)	2.4 (2.44)	–1.4 (0.34); –2.0 to –0.7	43	3.7 (2.51)	2.9 (3.19)	–0.9 (0.35); –1.6 to –0.2	–0.5 (0.49)	–1.4 to 0.5; *p*=0.352
EMO	45	3.2 (0.77)	1.4 (1.10)	–1.8 (0.18); –2.1 to –1.4	43	3.3 (0.87)	1.8 (1.31)	–1.5 (0.18); –1.9 to –1.1	–0.3 (0.26)	(–0.8 to 0.2); *p*=0.259
ESS	45	7.4 (3.52)	5.6 (3.84)	–2.4 (0.54); –3.5 to –1.3	43	9.4 (5.02)	6.4 (4.92)	–2.6 (0.56); –3.7 to –1.5	0.2 (0.79)	(–1.4 to 1.7); *p*=0.820
PDQ-8	45	11.2 (5.04)	7.1 (5.03)	–4.1 (0.60); –5.3 to –2.9	43	10.5 (5.07)	7.1 (5.03)	–3.5 (0.62); –4.7 to –2.3	–0.6 (0.87)	(–2.3 to 1.2); *p*=0.517

Unstructured covariance matrix, Kenward–Roger approximation for denominator degrees of freedom. ^*∗*^Adjusted with factors treatment, maintenance period, and covariate baseline. CI, confidence interval; EMO, early morning off; ESS, Epworth Sleepiness Scale; FAS, full analysis set; IR, immediate release; NHQ, Nocturnal Hypokinesia Questionnaire; PDQ-8, Parkinson's Disease Questionnaire-8; PDSS-2, Parkinson's Disease Sleep Scale 2^nd^ version; SCOPA-S, Scales for Outcomes in Parkinson's Disease-Sleep Scale; SD, standard deviation; SE, standard error; SR, sustained release.

**Table 4:** Overall summary of safety (TS).

	Pramipexole SR (*N* = 49)	Pramipexole IR (*N* = 49)	Total (*N* = 98)
AEs by category, *n* (%)			
Any AEs, *n* (%)	21 (42.9)	27 (55.1)	48 (49.0)
Severe AEs, *n* (%)	0 (0.0)	1 (2.0)	1 (1.0)
Drug-related AEs, *n* (%)	16 (32.7)	19 (38.8)	35 (35.7)
Serious AEs, *n* (%)	0 (0.0)	1 (2.0)	1 (1.0)
AEs leading to discontinuation, *n* (%)	1 (2.0)	1 (2.0)	2 (2.0)

AEs by PT^*∗*^, *n* (%)			
Dizziness	3 (6.1)	3 (6.1)	6 (6.1)
Nausea	2 (4.1)	2 (4.1)	4 (4.1)
Dyskinesia	1 (2.0)	3 (6.1)	4 (4.1)
Somnolence	1 (2.0)	3 (6.1)	4 (4.1)
Constipation	2 (4.1)	1 (2.0)	3 (3.1)
Upper respiratory tract infection	2 (4.1)	1 (2.0)	3 (3.1)
Oedema peripheral	1 (2.0)	2 (4.1)	3 (3.1)
Headache	1 (2.0)	2 (4.1)	3 (3.1)
Compulsive sexual behaviour	2 (4.1)	0 (0.0)	2 (2.0)
Dermatitis allergic	2 (4.1)	0 (0.0)	2 (2.0)
Orthostatic hypotension	2 (4.1)	0 (0.0)	2 (2.0)
Chest pain	0 (0.0)	2 (4.1)	2 (2.0)
Bradykinesia	0 (0.0)	2 (4.1)	2 (2.0)
Cough	0 (0.0)	2 (4.1)	2 (2.0)

A patient may be counted in more than one seriousness criterion. AE, adverse event; IR, immediate release; PT, preferred term; SR, sustained release; TS, treated set.  ^*∗*^With frequency >2% in either pramipexole groups; listed in descending order for total pramipexole.

## Data Availability

To ensure independent interpretation of clinical study results, Boehringer Ingelheim grants all external authors access to all relevant material, including participant-level clinical study data, and relevant material as needed by them to fulfill their role and obligations as authors under the ICMJE criteria. Furthermore, clinical study documents (e.g., study report, study protocol, and statistical analysis plan) and participant clinical study data are available to be shared after publication of the primary manuscript in a peer-reviewed journal and if regulatory activities are complete and other criteria met per the BI Policy on Transparency and Publication of Clinical Study Data: https://trials.boehringer-ingelheim.com/. Prior to providing access, documents will be examined, and, if necessary, redacted, and the data will be de-identified, to protect the personal data of study participants and personnel and to respect the boundaries of the informed consent of the study participants. Clinical study reports and related clinical documents can also be requested via the link https://trials.boehringer-ingelheim.com/. All requests will be governed by a Document Sharing Agreement. Bona fide, qualified scientific and medical researchers may request access to de-identified, analysable participant clinical study data with corresponding documentation describing the structure and content of the datasets. Upon approval, and governed by a Data Sharing Agreement, data are shared in a secured data-access system for a limited period of one year, which may be extended upon request. Researchers should use the https://trials.boehringer-ingelheim.com/link to request access to the study data.

## Supplementary Materials

Supplementary Materials The following tables are included in a supplementary file. Table S1: inclusion and exclusion criteria. Table S2: description of outcome measures for further efficacy endpoints. Table S3: adjusted mean changes in PDSS-2 total score at week 18 from baseline by subgroups (FAS). Table S4: adjusted mean changes in psychological health, quality of life, and caregiver burden at week 18 from baseline (FAS). Table S5: adjusted men changes in MDS-UPDRS IV items and CBI subscales at week 18 from baseline (FAS). Table S6: adjusted mean changes in MDS-UPDRS IV at week 18 from baseline by subgroups (FAS).
