# Supplementary material for: Efficacy and Safety of Pramipexole Sustained Release versus Immediate Release Formulation for Nocturnal Symptoms in Chinese Patients with Advanced Parkinson's Disease: A Pilot Study
Source: Parkinsons Dis. 2021 Mar 3;2021:8834950. doi: 10.1155/2021/8834950 (PMC7946461; doi:10.1155/2021/8834950)
Supplement: Supplementary Materials — The following tables are included in a supplementary file. Table S1: inclusion and exclusion criteria. Table S2: description of outcome measures for further efficacy endpoints. Table S3: adjusted mean changes in PDSS-2 total score at week 18 from baseline by subgroups (FAS). Table S4: adjusted mean changes in psychological health, quality of life, and caregiver burden at week 18 from baseline (FAS). Table S5: adjusted men changes in MDS-UPDRS IV items and CBI subscales at week 18 from baseline (FAS). Table S6: adjusted mean changes in MDS-UPDRS IV at week 18 from baseline by subgroups (FAS). [file 8834950.f1.docx]

## Efficacy and safety of pramipexole sustained release versus immediate release formulation for nocturnal symptoms in Chinese patients with advanced Parkinson’s disease: a pilot study

Haiyan Zhou^1+^, Shuhua Li^2,3+^, Hongmei Yu^4^, Shenggang Sun^5^, Xinhua Wan^6^, Xiaodong Zhu^7^, Chun-Feng Liu^8^, Ling Chen^9^, Wei Xiang^10^, Yaqing Sun^10^, Haibo Chen^2,3^*, Shengdi Chen^1^*; SUSTAIN study investigators

^1^ Department of Neurology, Ruijin Hospital, Shanghai Jiao Tong University School of Medicine, Shanghai, People’s Republic of China

^2^ Department of Neurology, Beijing Hospital, National Center of Gerontology, Beijing, People’s Republic of China

^3^ Department of Neurology, Institute of Geriatric Medicine, Chinese Academy of Medical Science, Beijing, People’s Republic of China

^4^ Department of Neurology, The First Hospital of China Medical University, Shenyang, People’s Republic of China

^5^ Department of Neurology, Wuhan Union Hospital, Wuhan, People’s Republic of China

^6^ Department of Neurology, Peking Union Medical College Hospital, Beijing, People’s Republic of China

^7^ Department of Neurology, Tianjin Medical University General Hospital, Tianjin, People’s Republic of China

^8^ Department of Neurology and Suzhou Clinical Research Center of Neurological Disease, The Second Affiliated Hospital of Soochow University, Suzhou, People’s Republic of China

^9^ Department of Neurology, The First Affiliated Hospital, Sun Yet-Sen University, Guangzhou, People’s Republic of China

^10^ Boehringer Ingelheim (China), Shanghai, People’s Republic of China

^+^ co-first authors

* co-corresponding authors

| **TABLE S1: Inclusion and exclusion criteria** |
| --- |
|  |
| **Inclusion criteria** |
| Patients fulfilling the following criteria were eligible for randomisation in the study: |
| - Advanced Parkinson’s disease (PD) confirmed by at least bradykinesia and/or signs of resting tremor and/or rigidity |
| - ≥ 2 years PD history |
| - ≥ 30 years of age at time of diagnosis |
| - Modified Hoehn and Yahr stage of 2 to 4 at on-time |
| - Clinically relevant sleep disturbances (i.e. PDSS-2 total score ≥ 18 at baseline) |
| - Discomfort at night due to inability to turn around in bed or move due to immobility |
| - Early morning off (i.e. the frequency of “feeling like bodily movements are poor when you wake up?” was at least 2 to 3 days during the past week) |
| - Motor fluctuations (at least 2 cumulative hours of off-time every day during waking hours, documented on a patient diary completed for 2 consecutive days before randomisation visit) |
| - Treatment with levodopa + (immediate or sustained release Levodopa/DDC inhibitor), or with a combination of levodopa and entacapone, at an optimised dose according to investigator’s judgment, stable for at least 4 weeks prior to randomisation visit. |
| - No treatment with a sustained release dopaminergic medication (i.e. sustained release Levodopa/DDC inhibitor) after supper, or any anti-PD medication after 9pm within 4 weeks prior to randomisation visit |
| - No treatment with dopamine agonists |
| - Concomitant treatment with one or more of the following medications was allowed (at a stable dose for at least 4 weeks prior to randomisation visit and with no intention by the investigator to change the treatment during the treatment phase): |
| - - Anti-parkinsonian anticholinergics |
| - - Selegiline, rasagiline, or other MAO-B-Inhibitor |
| - - Amantadine |
| - - Entacapone (or other COMT-Inhibitor) |
| - Women of childbearing potential (WOCBP)* and men able to father a child had to have been ready and able to use highly effective methods of birth control |
| - Signed and dated written informed consent |
| Exclusion criteria |
| Patients that met any of the following criteria were excluded from the trial: |
| - Secondary parkinsonian syndromes related to medication (e.g. metoclopramide, flunarizine), metabolic disorders (e.g. Wilson’s disease), encephalitis or degenerative diseases (e.g. progressive supranuclear palsy) |
| - Dementia, as defined by a Mini-Mental State Exam score <24 at screening visit |
| - Any psychiatric disorder according to DSM-V Diagnostic and Statistical Manual of Mental Disorders, 5th edition criteria that could prevent compliance or completion of the study and/or put the patient at risk if he/she took part in the study |
| - History of psychosis, except history of drug induced hallucinations (provided the investigator considers that participation in the trial would not represent a significant risk for the patient) |
| - History of deep brain stimulation |
| - History of nucleus lesioning |
| - Clinically significant electrocardiogram (ECG) abnormalities at screening visit, according to investigator’s judgement and/or symptomatic orthostatic hypotension (i.e. clinical symptoms of orthostatic hypotension associated with a decline ≥20 mmHg in SBP and a decline ≥10 mmHg in diastolic blood pressure (DBP), at 1 minute after standing compared with the previous supine systolic and DBP obtained after 5 minutes of quiet rest) at screening or randomisation visit |
| - Major surgery (major according to the investigator’s assessment) performed within12 weeks prior to randomisation or planned within 12 months after screening, e.g. hip replacement |
| - Any other clinically significant disease, whether treated or not, that could put the patient at risk or could prevent compliance or completion of the study |
| - Serious Sleep Apnea Hypopnea Syndrome (i.e. the scoring of question 15 in PDSS-2 ≥3, that meant frequency was at least 4 to 5 days during the past week) |
| - Any documented active or suspected malignancy or history of malignancy within 5 years prior to screening, except appropriately treated basal cell carcinoma of the skin or in situ carcinoma of uterine cervix |
| - Serum levels of AST (SGOT), ALT (SGPT), alkaline phosphatases or total bilirubin - >2 ULN (on screening lab test) |
| - Creatinine clearance < 50 mL/min (estimated by the local lab / the investigator using the Modification of Diet in Renal Disease (MDRD), and calculated on screening lab test) |
| - Any hypnotic medication within 4 weeks prior to the randomisation visit |
| - Any medication (including intra-muscular formulations) with central dopaminergic antagonist activity within 4 weeks prior to the randomisation visit (i.e. typical neuroleptics, atypical antipsychotics, reserpine, methyldopa, centrally-active antiemetics,etc.) |
| - Treatment with methylphenidate and/or cinnarizine and/or amphetamines within 4 weeks prior to randomisation visit |
| - Treatment with flunarizine within 3 months prior to randomisation visit |
| - Known hypersensitivity to Pramipexole or its excipients |
| - Women who were pregnant, nursing, or who planned to become pregnant in the trial |

*A woman is considered of childbearing potential (WOCBP), i.e. fertile, following menarche and until becoming post-menopausal unless permanently sterile. Permanent sterilization methods include hysterectomy, bilateral salpingectomy and bilateral oophorectomy. Tubal ligation is NOT a method of permanent sterilization. A postmenopausal state is defined as no menses for 12 months without an alternative medical cause.

PDSS-2, Parkinson’s Disease Sleep Scale 2^nd^ version; DDC, Dopa-Decarboxylase; MAO-B, monoamine oxidase type B; COMT, Catechol-O-Methyltransferase

## SUPPLEMENTARY MATERIAL

TABLE S2: Description of outcome measures for further efficacy endpoints

| **Outcome measures** | **Description** |
| --- | --- |
| **Further efficacy endpoints:** | |
| Movement Disorder Society-sponsored revision of the Unified Parkinson’s disease Rating Scale (MDS-UPDRS) part II | MDS-UPDRS Part II (Motor Aspects of Experiences of Daily Living) consists of 13 activities, each of which is scored from 0 (normal) to 4 (severe parkinsonian symptoms). Based on the patient’ status over the previous week before each visit, each of these 13 activities was scored. Part II of the MDS-UPDRS does not have separate on or off ratings. A reduction in the MDS-UPDRS part II score over time corresponds to an improvement in activities of daily living.  The various items are the following: speech, saliva & drooling, chewing and swallowing, eating tasks, dressing, hygiene, handwriting, doing hobbies and other activities, turning in bed, tremor, getting out of fed or a car or a deep chair, walking and balance, freezing. |
| Movement Disorder Society-sponsored revision of the Unified Parkinson’s disease Rating Scale (MDS-UPDRS) part IV | MDS-UPDRS Part IV (Motor Complications) consists of 6 questions, each of which is scored from 0 (normal) to 4 (severe). Based on the patient’ status over the past week before each visit, each of these 6 questions was to be scored. A reduction in the MDS-UPDRS part IV score over time corresponds to an improvement in motor complications.  The various items are the following: time spent with dyskinesia, functional impact of dyskinesia, time spent in the off state, functional impact of fluctuations, complexity of motor fluctuations, painful off-state dystonia. |
| Geriatric Depression Scale (GDS)-15 | The GDS-15 is a widely used instrument for depression screening in the general geriatric population and in PD as well. Because it is brief, no somatically focused, and can be observer – or self- administered, the GDS-15 offers promise as a practical, valid rating instrument for PD in the clinical care as well as for research purposes. The GDS-15 consists of 15 yes/no questions, with total score ranging from 0 (without depression) to 15 (very severe depression). At each question, patients had to circle the answer (yes or no) that best describes how patients felt over the past week. A score of ≥ 5 suggests depression. |
| EQ-5D-5L | The EQ-5D-5L is a standardized health-related quality of life questionnaire developed by the EuroQoL Group in order to provide a simple, generic measure of health for clinical and economic appraisal. It is applicable to a wide range of health conditions and treatments, and provides a simple descriptive profile and a single index value for health status. The EQ-5D-5L consists of 2 pages: a 5-item patient reported descriptive measure and a visual analogue scale. It produces a numeric score for health status on which full health has a value of 100 and death has a value of 0. The descriptive measure comprises 5 dimensions: mobility, self-care, usual activity, pain/discomfort, and anxiety/depression. Patients were to complete the scale based on their status on the day of the visit. |

TABLE S3: Adjusted mean changes in PDSS-2 total score at week 18 from baseline by subgroups (FAS)

| Subgroup | Pramipexole SR | | | | | Pramipexole IR | | | | SR vs IR | |
| --- | --- | --- | --- | --- | --- | --- | --- | --- | --- | --- | --- |
|  | N | Baseline, mean | Week 18, mean | Change*, mean | N | | Baseline, mean | Week 18, mean | Change*, mean | Adjusted mean difference | p-value |
| Age, years |  |  |  |  |  | |  |  |  |  |  |
| <65 | 25 | 27.0 | 15.0 | -12.9 | 29 | | 29.2 | 13.0 | -15.5 | 2.6 | 0.271 |
| ≥65 | 20 | 29.4 | 14.1 | -14.9 | 14 | | 29.6 | 17.1 | -12.0 | -2.9 | 0.223 |
| Pramipexole final dose, mg/d | | | | | | | | | | | |
| Low (0.375-1.5) | 32 | 27.3 | 14.1 | -13.5 | 34 | | 28.8 | 14.1 | -14.0 | 0.5 | 0.819 |
| Medium (2.25-3.0) | 13 | 29.8 | 15.8 | -14.4 | 9 | | 31.4 | 15.0 | -15.7 | 1.3 | 0.684 |
| Hoehn & Yahr stage on-phase | | | | | | | | | | | |
| Stage 2 | 37 | 26.9 | 14.0 | -13.1 | 30 | | 28.1 | 14.8 | -12.6 | -0.4 | 0.834 |
| Stage 2.5 | 4 | 30.0 | 16.5 | -13.5 | 8 | | 30.1 | 13.1 | -17.0 | 3.4 | 0.337 |
| Stage 3 | 4 | 36.3 | 18.0 | -18.6 | 5 | | 35.8 | 13.2 | -22.4 | 3.8 | 0.514 |
| ADL severity at baseline | | | | | | | | | | | |
| Low (MDS-UPDRS II ≤12) | 10 | 25.7 | 14.6 | -12.0 | 7 | | 28.6 | 10.3 | -16.9 | 4.9 | 0.208 |
| High (MDS-UPDRS II >12) | 35 | 28.7 | 14.6 | -14.2 | 36 | | 29.5 | 15.1 | -14.0 | -0.3 | 0.890 |
| Complication severity at baseline | | | | | | | | | | | |
| Low (MDS-UPDRS IV ≤4) | 6 | 26.7 | 17.2 | -9.3 | 7 | | 26.0 | 16.0 | -8.6 | -0.7 | 0.887 |
| High (MDS-UPDRS IV >4) | 39 | 28.3 | 14.2 | -14.5 | 36 | | 30.0 | 14.0 | -15.4 | 0.9 | 0.625 |
| Disease severity at baseline, hours | | | | | | | | | | | |
| High (off-time from patient diary ≥4) | 45 | 28.0 | 14.6 | -13.7 | 43 | | 29.3 | 14.3 | -14.4 | 0.7 | 0.688 |
| Baseline L-dopa dose, mg | | | | | | | | | | | |
| ≤400 | 32 | 28.6 | 15.2 | -13.2 | 20 | | 28.7 | 14.8 | -13.9 | 0.7 | 0.780 |
| >400 | 13 | 26.8 | 13.0 | -15.4 | 23 | | 30.0 | 14.0 | -14.8 | -0.6 | 0.797 |
| Baseline equivalent L-dopa dose, mg | | | | | | | | | | | |
| ≤400 | 16 | 29.4 | 15.1 | -13.6 | 12 | | 28.5 | 13.8 | -15.0 | 1.4 | 0.683 |
| >400 | 29 | 27.3 | 14.3 | -13.9 | 31 | | 29.7 | 14.5 | -14.0 | 0.1 | 0.962 |

Unstructured covariance matrix, Kenward-Roger approximation for denominator degrees of freedom.

*Adjusted with factors treatment, maintenance period and covariate baseline.

ADL, Activities of Daily Living; FAS, full analysis set; IR, immediate release; PD, Parkinson’s Disease; PDSS, Parkinson’s Disease Sleep Scale-2; SR, sustained release.

TABLE S4: Adjusted mean changes in psychological health, quality of life and caregiver burden at week 18 from baseline (FAS)

|  | Pramipexole SR | | | | Pramipexole IR | | | | | SR vs IR | |
| --- | --- | --- | --- | --- | --- | --- | --- | --- | --- | --- | --- |
|  | N | Baseline, mean (SD) | Week 18, mean (SD) | Change, mean (SE); 95% CI | N | Baseline, mean (SD) | Week 18, mean (SD) | Change, mean (SE); 95% CI | Adjusted* mean difference (SE) | | 95% CI, p-value |
| MDS-UPDRS II | 45 | 16.5 (5.88) | 10.9 (6.25) | –5.8 (0.86); –7.5 to –4.1 | 43 | 17.4 (5.74) | 12.5 (7.48) | –4.7 (0.89); –6.5 to –3.0 | –1.1 (1.25) | | –3.5 to 1.4 ; p = 0.395 |
| MDS-UPDRS IV | 45 | 7.2 (2.78) | 3.7 (2.50) | –3.4 (0.35); –4.1 to –2.7 | 43 | 6.6 (2.66) | 4.5 (2.68) | –2.3 (0.36); –3.0 to –1.6 | –1.1 (0.51) | | –2.1 to –0.1; p = 0.036 |
| GDS-15 | 45 | 6.8 (3.82) | 4.6 (3.06) | –2.3 (0.42); –3.1 to –1.4 | 43 | 7.0 (3.92) | 5.7 (3.86) | –1.2 (0.43); –2.1 to –0.4 | –1.1 (0.61) | | –2.3 to 0.2 ; p = 0.086 |
| EQ-5D-5L | 45 | 0.6 (0.22) | 0.8 (0.16) | 0.2 (0.02); 0.1 to 0.2 | 43 | 0.6 (0.23) | 0.8 (0.18) | 0.2 (0.02); 0.1 to 0.2 | 0.01 (0.03) | | –0.06 to 0.07 ; p = 0.885 |
| CBI | 13 | 28.8 (24.23) | 19.0 (18.51) | –12.3 (3.57); –19.6 to –4.9 | 11 | 34.5 (20.32) | 28.5 (15.06) | –3.2 (3.93); –11.3 to 4.9 | –9.0 (5.33) | | –20.0 to 1.9; p = 0.102 |

Unstructured covariance matrix, Kenward-Roger approximation for denominator degrees of freedom.

*Adjusted with factors treatment, maintenance period and covariate baseline.

CBI, Caregiver Burden Index; CI, confidence interval; FAS, full analysis set; GDS, Geriatric Depression Scale; IR, immediate release; MDS-UPDRS, Movement Disorder Society-sponsored revision of the Unified Parkinson’s disease Rating Scale; SD, standard deviation; SE, standard error; SR, sustained release.

TABLE S5: Adjusted men changes in MDS-UPDRS IV items and CBI subscales at week 18 from baseline (FAS)

|  | Pramipexole SR | | | | | Pramipexole IR | | | | | SR vs IR | |
| --- | --- | --- | --- | --- | --- | --- | --- | --- | --- | --- | --- | --- |
|  | N | Baseline, mean (SD) | Week 18, mean (SD) | Change, mean (SE); 95% CI | N | | Baseline, mean (SD) | Week 18, mean (SD) | Change, mean (SE); 95% CI | Adjusted* mean difference (SE) | | 95% CI, p-value |
| MDS-UPDRS IV items | | | | | | | | | | | | |
| 4.1 Time spent with dyskinesias | 45 | 0.4 (0.69) | 0.2 (0.40) | –0.2 (0.06); –0.3 to –0.1 | 43 | | 0.3 (0.47) | 0.4 (0.50) | 0.1 (0.06); –0.1 to 0.2 | –0.3 (0.08) | | –0.4 to –0.1; p = 0.003 |
| 4.2 Functional impact of dyskinesia | 45 | 0.4 (0.87) | 0.2 (0.50) | –0.3 (0.07); –0.4 to –0.1 | 43 | | 0.5 (1.08) | 0.4 (0.69) | –0.1 (0.07); –0.3 to 0.0 | –0.1 (0.10) | | –0.3 to 0.1; p = 0.174 |
| 4.3 Time spent in the “off” state | 45 | 1.9 (0.79) | 1.0 (0.64) | –0.9 (0.09); –1.1 to –0.7 | 43 | | 1.8 (0.78) | 1.1 (0.70) | –0.8 (0.10); –1.0 to –0.6 | –0.1 (0.14) | | –0.4 to 0.2; p = 0.435 |
| 4.4 Funtional impact of fluctuations | 45 | 1.9 (0.87) | 1.0 (0.98) | –1.0 (0.14); –1.3 to –0.7 | 43 | | 2.0 (0.98) | 1.2 (1.06) | –0.8 (0.14); –1.0 to –0.5 | –0.2 (0.20) | | –0.6 to 0.2; p = 0.286 |
| 4.5 Complexity of motor fluctuations | 45 | 1.2 (0.68) | 0.8 (0.44) | –0.4 (0.06); –0.5 to –0.3 | 43 | | 1.1 (0.37) | 0.9 (0.37) | –0.3 (0.06); –0.4 to –0.1 | –0.1 (0.09) | | –0.3 to 0.1; p = 0.227 |
| 4.6 Painful “off” state dystonia | 45 | 1.3 (1.41) | 0.5 (1.06) | –0.6 (0.15); –0.9 to –0.3 | 43 | | 0.8 (1.08) | 0.5 (1.10) | –0.5 (0.16); –0.8 to –0.2 | –0.2 (0.22) | | –0.6 to 0.3; p = 0.496 |
| CBI subscales | | | | | | | | | | | | |
| Time Dependence | 13 | 9.8 (6.82) | 7.0 (6.77) | –3.9 (1.38); –6.7 to –1.1 | 11 | | 13.2 (7.40) | 10.5 (5.96) | –1.5 (1.52); –4.6 to 1.6 | –2.4 (2.09) | | –6.7 to 1.9; p = 0.263 |
| Emotional Burden | 13 | 2.0 (4.40) | 1.5 (2.57) | –0.8 (0.57); –2.0 to 0.4 | 11 | | 2.6 (3.47) | 2.3 (2.61) | –0.3 (0.63); –1.6 to 1.0 | –0.5 (0.85) | | –2.3 to 1.2; p = 0.538 |
| Developmental | 13 | 7.2 (6.56) | 5.4 (5.24) | –2.0 (1.01); –4.1 to 0.0 | 11 | | 6.6 (4.37) | 6.6 (4.11) | 0.1 (1.11); –2.2 to 2.4 | –2.1 (1.50) | | –5.2 to 1.0 p = 0.170 |
| Social Burden | 13 | 3.6 (4.17) | 2.2 (2.70) | –1.7 (0.77); –3.3 to –0.2 | 11 | | 4.4 (4.76) | 3.2 (3.09) | –0.5 (0.84); –2.2 to 1.3 | –1.3 (1.14) | | –3.7 to 1.1; p = 0.272 |
| Physical Burden | 13 | 6.3 (6.02) | 2.9 (3.07) | –3.7 (0.68); –5.0 to –2.3 | 11 | | 7.6 (4.37) | 5.9 (2.98) | –1.0 (0.75); –2.6 to 0.5 | –2.6 (1.02) | | –4.7 to –0.5; p = 0.016 |

Unstructured covariance matrix, Kenward-Roger approximation for denominator degrees of freedom.

*Adjusted with factors treatment, maintenance period and covariate baseline.

CBI, Caregiver Burden Index; CI, confidence interval; FAS, full analysis set; IR, immediate release; MDS-UPDRS, Movement Disorder Society-sponsored revision of the Unified Parkinson’s disease Rating Scale; SD, standard deviation; SE, standard error; SR, sustained release.

TABLE S6: Adjusted mean changes in MDS-UPDRS IV at week 18 from baseline by subgroups (FAS)

| Subgroup | Pramipexole SR | | | | Pramipexole IR | | | | SR vs IR | |
| --- | --- | --- | --- | --- | --- | --- | --- | --- | --- | --- |
|  | N | Baseline, mean (SD) | Week 18, mean (SD) | Change, mean (SE); 95% CI | N | Baseline, mean (SD) | Week 18, mean (SD) | Change, mean (SE); 95% CI | Adjusted* mean difference (SE) | 95% CI, p-value |
| Age, years | | | | | | | | | | |
| <65 | 25 | 7.2 (2.72) | 3.4 (2.64) | –3.6 (0.49); –4.6 to –2.6 | 29 | 6.5 (1.84) | 4.3 (2.51) | –2.4 (0.46); –3.3 to –1.5 | –1.2 (0.68) | –2.6 to 0.1; p = 0.080 |
| ≥65 | 20 | 7.3 (2.92) | 4.2 (2.28) | –3.2 (0.51); –4.2 to –2.1 | 14 | 6.7 (3.93) | 4.8 (3.09) | –2.1 (0.61); –3.4 to –0.9 | –1.0 (0.79) | –2.7 to 0.6; p = 0.202 |
| PD duration, years | | | | | | | | | | |
| 2-<5 | 31 | 7.2 (3.01) | 3.5 (2.51) | –3.5 (0.43); –4.4 to –2.7 | 25 | 6.4 (2.14) | 4.3 (2.50) | –2.4 (0.48); –3.4 to –1.1 | –1.1 (0.65) | –2.4 to 0.2; p = 0.096 |
| ≥5 | 14 | 7.3 (2.27) | 4.2 (2.49) | –3.2 (0.62); –4.4 to –1.9 | 18 | 6.8 (3.30) | 4.7 (2.99) | –2.2 (0.56); –3.4 to –1.1 | –0.9 (0.83) | –2.6 to 0.8; p = 0.271 |
| Baseline equivalent L-dopa dose, mg | | | | | | | | | | |
| ≤400 | 16 | 7.6 (2.73) | 3.4 (2.73) | –3.8 (0.64); –5.1 to –2.5 | 12 | 6.2 (1.53) | 4.3 (2.45) | –2.5 (0.75); –4.1 to –1.0 | –1.3 (1.01) | –3.4 to 0.8; p = 0.211 |
| >400 | 29 | 7.0 (2.83) | 3.9 (2.40) | –3.2 (0.43); –4.0 to –2.3 | 31 | 6.7 (2.99) | 4.6 (2.80) | –2.3 (0.42); –3.1 to –1.4 | –0.9 (0.60) | –2.1 to 0.3; p = 0.137 |
| Presence of dyskinesia at baseline | | | | | | | | | | |
| No dyskinesia | 31 | 6.3 (2.14) | 3.3 (2.11) | –2.8 (0.42); –3.6 to –1.9 | 29 | 5.5 (1.82) | 4.0 (2.46) | –2.0 (0.43); –2.8 to –1.1 | –0.8 (0.61) | –2.0 to 0.4; p = 0.192 |
| Any dyskinesia | 14 | 9.3 (2.95) | 4.8 (3.02) | –4.5 (0.58); –5.7 to –3.3 | 14 | 8.9 (2.71) | 5.5 (2.93) | –3.4 (0.58); –4.6 to –2.2 | –1.1 (0.83) | –2.8 to 0.6; p = 0.210 |
| Time spent in the off state at baseline^+^ | | | | | | | | | | |
| Slight | 16 | 5.9 (2.19) | 4.2 (2.56) | –2.1 (0.71); –3.6 to –0.6 | 11 | 5.9 (2.74) | 3.5 (2.81) | –2.4 (0.85); –4.2 to –0.6 | 0.3 (1.11) | –2.0 to 2.6; p = 0.795 |
| Mild | 17 | 6.8 (2.05) | 3.1 (2.05) | –3.6 (0.50); –4.6 to –2.6 | 22 | 6.5 (1.57) | 4.2 (2.02) | –2.4 (0.44); –3.3 to –1.5 | –1.2 (0.66) | –2.5 to 0.2; p = 0.089 |
| Moderate | 12 | 9.6 (3.03) | 4.1 (2.97) | –5.5 (0.52); –6.6 to –4.4 | 8 | 9.0 (3.12) | 7.4 (2.33) | –1.8 (0.64); –3.1 to –0.4 | –3.7 (0.82) | –5.5 to –2.0; p = 0.000 |

Unstructured covariance matrix, Kenward-Roger approximation for denominator degrees of freedom. According to MDS-UPDRS IV, presence of dyskinesia is defined according to item 4.1 as 0 point for “no dyskinesia” or > 0 points for “any dyskinesia”; time spent in the “off” state is defined according to item 4.3 as 0 point for “normal” (no off time), 1 point for “slight” (≤25% of waking day), 2 points for “mild” (26–50% of waking day), 3 points for “moderate” (51–75% of waking day), or 4 points for “severe” (>75% of waking day).

*Adjusted with factors treatment, maintenance period and covariate baseline.

^+^No patients reported “none” (item 4.3 = 0) or “severe” amount of off state hours (item 4.3 = 4);two patients in the IR group reported “normal .

CI, confidence interval; IR, immediate release; FAS, full analysis set; MDS-UPDRS, Movement Disorder Society-sponsored revision of the Unified Parkinson’s disease Rating Scale; SD, standard deviation; SE, standard error; SR, sustained release.
